# Anhedonia Links Sleep Problems and Suicidal Thoughts: An Intensive Longitudinal Study in High-Risk Adolescents

**DOI:** 10.1007/s10802-024-01275-w

**Published:** 2024-12-16

**Authors:** Kinjal K. Patel, Jaclyn C. Kearns, Dan Foti, Wilfred R. Pigeon, Evan M. Kleiman, Catherine R. Glenn

**Affiliations:** 1 Department of Psychology, Old Dominion University, Norfolk, VA USA; 2 Virginia Consortium Program in Clinical Psychology, Norfolk, VA USA; 3 VA Boston Healthcare System, Behavioral Science Division of the National Center for PTSD, Boston, MA USA; 4 Department of Psychiatry, Boston University Chobanian & Avedisian School of Medicine, Boston, MA USA; 5 Department of Psychological Sciences, Purdue University, West Lafayette, IN USA; 6 Department of Psychiatry, University of Rochester Medical Center, Rochester, NY USA; 7 US Department of Veterans Affairs, Center of Excellence for Suicide Prevention, Finger Lakes Health Care System, Canandaigua, NY USA; 8 Department of Psychology, Rutgers University, The State University of New Jersey, New Brunswick, NJ USA

**Keywords:** Adolescent, Sleep problems, Anhedonia, Suicidal thoughts, Ecological momentary assessment

## Abstract

Growing research indicates that sleep problems are a robust independent risk factor for suicidal thoughts and behaviors among youth. However, relatively little is known about *how* this risk is conferred. This study used an intensive longitudinal design to investigate anhedonia as a mechanism linking sleep problems and next-day suicidal thoughts in a clinically high-risk sample of adolescents. Adolescents (*N* = 48; *M*_age_=14.96; 77.1% white, 64.6% female) completed an ecological momentary assessment (EMA) study design for 28 days following discharge from acute psychiatric care for suicide risk. Daily sleep diaries were used to assess prior night total sleep time and sleep onset latency. Ecological momentary assessment was used to assess anhedonia and suicidal thoughts up to six times per day. A series of multi-level structural equation models were used to examine facets of anhedonia as parallel mediators of the association between sleep problems and next-day suicidal thoughts. Significant direct effects were found between sleep problems and consummatory anhedonia, consummatory anhedonia and suicidal thoughts, and anticipatory anhedonia and suicidal thoughts. There were significant indirect (mediated) effects between sleep problems and next-day suicidal thoughts through consummatory anhedonia, but not anticipatory anhedonia. Findings provide initial evidence as to *how* sleep problems may confer risk for next-day suicidal thoughts– by increasing consummatory anhedonia. Future research is needed to replicate these findings in larger samples and investigate how modifying anhedonia may mitigate suicide risk in youth.

## Introduction

Suicidal thoughts and behaviors (STBs) are a major public health concern among youth. Prevalence rates from a 2023 nationwide survey indicate that, in the past year, 20% of youth seriously considered suicide and 9% of youth made a suicide attempt (Centers for Disease Control and Prevention [CDC], 2023b). Moreover, emerging research indicates an escalation of STBs (e.g., transitions from thinking about suicide to planning or attempting suicide, Nock et al., 2013) during adolescence. These high rates are alarming as STBs during adolescence are associated with significant interpersonal and health impairment, both concurrently (Glenn & Klonsky, 2013) and into adulthood (Copeland et al., 2017). Taken together, adolescence represents a critical window of opportunity for early detection, intervention, and prevention of STBs (King et al., 2018; Whitlock et al., 2014; Wyman, 2014).

Most studies to date have focused on identifying distal risk factors for STBs, distinguishing *who* is at risk for STBs. However, recent meta-analyses indicate that distal risk factors are unable to distinguish the *when* or timing of that risk (Franklin et al., 2017; Ribeiro et al., 2016), limiting our ability to intervene and reduce risk for STBs. Identifying proximal, time-varying, and modifiable risk factors may yield key information about when individuals may be at greatest risk for STBs and how we can intervene to reduce risk (Kleiman et al., 2019). Doing so requires techniques that allow for intensive longitudinal, real-time monitoring over shorter time intervals. Ecological momentary assessment (EMA) may be helpful to employ since this methodology allows for the examination of within-person, day-to-day fluctuations in individuals’ experiences by collecting data multiple times each day over short data collection intervals (Shiffman et al., 2008). In addition to examining day-to-day and within-day fluctuations in STBs (e.g., how suicidal thoughts change), real-time monitoring approaches, like EMA, may be particularly useful for identifying proximal factors *for* STBs (Kleiman et al., 2017). One promising proximal, time-varying risk factor for STBs is sleep problems.

### Sleep Problems and STBs

Sleep problems refer to a broad range of difficulties associated with the onset and maintenance of sleep, and growing research indicates a robust association between sleep problems and STBs (Kearns et al., 2020; Liu et al., 2020a; McCall & Black, 2013; Pigeon et al., 2012, 2016). Commonly endorsed sleep problems among adults and adolescents include frequent difficulties falling asleep (i.e., longer sleep onset latency [SOL]) or staying asleep, which often results in insufficient sleep duration, or shorter total sleep time (TST; American Psychiatric Association, 2013). Notably, sleep problems are a modifiable risk factor for STBs because they are amenable to clinical intervention (Blake et al., 2017, 2018; Taylor & Pruiksma, 2014).

Sleep problems and STBs have been consistently linked among adults in both cross-sectional and longitudinal studies (Bernert et al., 2005; Golding et al., 2015; Krakow et al., 2011; Pigeon et al., 2012, 2016; Russell et al., 2019). Emerging research in adults has found that sleep problems predict short-term increases in suicidal thoughts over seven-day follow-up (Bernert et al., 2017) or next-day (Cox et al., 2023; Littlewood et al., 2019).

Although far less research has examined sleep problems in youth, some studies have found sleep problems to be cross-sectional correlates and long-term predictors (i.e., over years) of STBs (Asarnow et al., 2020; Goldstein et al., 2008; Kearns et al., 2020; Russell et al., 2018). One study with high-risk adolescents recently discharged from acute psychiatric care for suicide risk who completed a 28-day EMA protocol found an association between certain sleep problems (i.e., nightmares, greater self-reported difficulties falling asleep) and next-day suicidal thoughts (Glenn et al., 2021). In another high-risk sample of adolescents and young adults (ages 13–23), Hamilton et al. (2023) demonstrated that actigraphy-estimated shorter TST was indirectly linked with next-day suicidal thoughts via affective reactivity to interpersonal events. Taken together, sleep problems show initial promise as a significant proximal, time-varying, and modifiable risk factor for suicidal thoughts in youth that warrants future study.

Several limitations of the extant research base remain to be addressed. First, far less is known about the sleep problem-STB link in youth. This is particularly critical, given the ongoing changes with sleep patterns and circadian rhythm that occur during adolescence (Harvey et al., 2018; McGlinchey & Harvey, 2015), which may further increase vulnerability to risk factors for STBs. Adolescents’ preference for a delayed sleep-wake schedule conflicts directly with early school start times, and often results in adolescents experiencing insufficient sleep duration (i.e., shorter TST) or sleep deprivation. In fact, most adolescents do not get the recommended 8–10 h of sleep per night (Galland et al., 2018; Hirshkowitz et al., 2015). Furthermore, adolescents may attempt to initiate sleep at a time that mismatches their circadian rhythm preference, resulting in difficulties falling asleep (i.e., longer SOL).

Second, there is a paucity of research that has examined the short-term links between sleep problems and STBs. Most existing studies are limited by their retrospective study design and temporally insensitive methodology restricting our understanding of the short-term relation between sleep problems and STBs. Given the fluctuating nature of both sleep problems (Becker et al., 2017) and STBs (Kleiman et al., 2017), shorter time intervals for measurement, using more temporally sensitive approaches, may yield more reliable information regarding the nature of this relationship which would further our understanding of *when* STB risk is greatest.

Lastly, although existing research has linked sleep problems and STBs (Kearns et al., 2020; Liu et al., 2020a; Pigeon et al., 2012, 2016), minimal research has focused on explicating the *mechanisms* underlying this relation, which could inform targets for intervention and prevention of STBs. One potential mechanism with substantive support that is hypothesized to link sleep problems and suicidal thoughts is anhedonia. Specifically, separate bodies of literature provide compelling evidence linking sleep problems and anhedonia, and anhedonia and STBs (reviewed separately next). To date, no studies in adolescents have examined this mechanistic relationship in one integrative model, hindering our understanding of *how* risk for STBs increases following sleep disruption.

### Anhedonia as a Potential Mechanism

Anhedonia refers to the diminished capacity to experience pleasure or interest from what would typically be a pleasurable event or activity. Theoretical models (Klein, 1987; Shankman et al., 2014), behavioral evidence from preclinical models (e.g., Berridge & Robinson, 2003), and clinical application in humans (Baskin-Sommers & Foti, 2015; Gilbert & Wilson, 2007; Treadway & Zald, 2011) suggest that anhedonia can be divided into anticipatory and consummatory components. Anticipatory anhedonia refers to the diminished capacity to experience pleasure from the expectation of reward, whereas consummatory anhedonia refers to the diminished capacity to experience pleasure from in-the-moment consumption (Treadway & Zald, 2011). Consistent evidence for these two facets of anhedonia comes from growing research related to clinical phenomena such as depression (Pizzagalli et al., 2009; Treadway & Zald, 2011). Specifically, prior studies have uncovered differential neurobiological and behavioral components of anhedonia in the context of depression, underscoring the utility of examining associations between these distinct aspects of anhedonia and clinical outcomes such as STBs (Pizzagalli et al., 2009; Treadway & Zald, 2011). Notably, recent real-time monitoring investigations into the time-varying nature of anhedonia similarly delineate anhedonia into anticipatory and consummatory phases (Bakker et al., 2017; Wieman et al., 2022; Wu et al., 2017).

### Sleep and Anhedonia

Decades of research have demonstrated the inextricable link between sleep and affective experiences (Ong et al., 2017; Palmer et al., 2023; Tomaso et al., 2020). Specifically, sleep problems are associated with lower positive affect (Cousins et al., 2011; Kalmbach et al., 2014; McCrae et al., 2008), often used as a proxy for anhedonia (Heininga et al., 2019). Further, sleep problems have also been linked to general anhedonia in adults (Kalmbach et al., 2017; Wieman et al., 2022) and adolescents (Burani et al., 2021; Casement et al., 2016). Notably, one daily diary study in undergraduate students demonstrated that shorter TST and poorer sleep quality predicted blunted next-day anticipatory *and* consummatory responses to reward (Wieman et al., 2022).

### Anhedonia and STBs

The consistent relationship between anhedonia and STBs is well-established in adults (Ballard et al., 2017; Bonanni et al., 2019; Loas et al., 2018; Winer et al., 2014, 2016; Yang et al., 2020b) and adolescents (Auerbach et al., 2015; Nock & Kazdin, 2002; Yang et al., 2021). A recent meta-analysis demonstrated an association between general anhedonia and suicidal thoughts, independent of total depression symptom severity (Ducasse et al., 2018). More recent studies have examined associations between anticipatory and consummatory components of anhedonia and suicidal thoughts. In a cross-sectional study of adult physicians, Loas et al. (2018) demonstrated a link between anticipatory anhedonia, but not consummatory anhedonia, and suicidal thoughts. In a longitudinal investigation, Yang et al. (2020a) found that anticipatory and consummatory anhedonia both mediated the link between stress and suicidal thoughts. In one cross-sectional study that examined anhedonia and risk for current suicidal thoughts among adults with insomnia, unlike lifelong or trait-level general anhedonia, only recent change of anhedonia severity was a risk factor for current suicidal thoughts (Dosogne et al., 2022). Collectively, extant research supports a robust association between anhedonia and STBs, and importantly these associations exist independently from depression.

Prior research supports anhedonia as a putative transdiagnostic factor associated with sleep problems and STBs and is indicative of a potential pathway linking sleep problems and STBs. Consistent with emerging theories examining associations between sleep problems and psychopathology broadly, it may be that sleep problems disrupt reward processing pathways resulting in increased anhedonia (i.e., greater difficulty experiencing or predicting pleasure from future activities), ultimately increasing suicidal thought intensity (Tubbs et al., 2022). However, to date, no studies have investigated anhedonia as a mechanism in the sleep-STB association in one integrative model.

## Current Study

The current study is a secondary data analysis of a prior study (Glenn et al., 2021)^1^. The current study builds and extends this prior study by testing anhedonia as a specific mechanism (mediator) linking sleep problems and suicidal thoughts in high-risk adolescents using a real-time monitoring approach.

We hypothesized that greater difficulties falling asleep (i.e., longer SOL) and shorter, or insufficient, sleep duration (i.e., shorter TST) would be associated with greater anhedonia. Next, we hypothesized that greater anhedonia would predict greater suicidal thoughts. Finally, we hypothesized that greater anhedonia would partially mediate the association between sleep problems and suicidal thoughts. Based on prior research, it is evident that examining specific facets of anhedonia is warranted, and as such, we aimed to parse anhedonia (i.e., anticipatory and consummatory) to understand differential mechanistic links that may exist in the sleep-anhedonia-STB association. Given the mixed findings from prior research, we did not have specific hypotheses about which facet of anhedonia would be a stronger mediator.

## Method

This study was part of a larger project examining short-term risk for STBs in adolescents during the high-risk period following discharge from acute psychiatric care. The method for this project has been described in detail in a prior paper (Glenn et al., 2022b). A brief overview of methods related to the current study are summarized below.

### Participants

The study sample consisted of 48 adolescents (ages 12–18, *M* = 14.96, *SD* = 1.60), who were eligible for the study if they had recently received acute psychiatric care (i.e., psychiatric emergency department, inpatient unit, or partial hospitalization) for suicide risk (i.e., suicide ideation with intent and/or plan, suicide attempt) and were being discharged to outpatient psychiatric care at the affiliated medical center. Participants self-identified their race and ethnicity as follows: Black or African American (*n* = 4, 8.3%), White (*n* = 37, 77.1%), Multi-racial (*n* = 5, 10.4%), Other/Do not wish to answer (*n* = 2, 4.17%), and Hispanic/Latinx (*n* = 6, 12.5%). Participants self-identified their gender identity as follows: cisgender female (*n* = 34, 64.6%), cisgender male (*n* = 9, 16.7%), and nonbinary or gender diverse (*n* = 10, 18.8%). Youth were not eligible to participate if they were unable to provide informed consent for themselves (e.g., due to extreme cognitive impairment, current mania or psychosis), unwilling to complete the study procedures (i.e., unwilling to wear wrist actigraphy device or complete smartphone-based EMA surveys), or a safety concern (i.e., imminent risk for suicide or other-directed violence). Of note, adolescents without a smartphone were loaned an Android (Tracfone) with a pre-paid data plan.

### Procedure

Adolescents were enrolled in the study within two weeks of discharge from acute psychiatric care. Prior to study participation, informed consent was obtained from the adolescent (assent if 12-17-years-old, consent if 18-years-old) and one parent or legal guardian (parental permission or consent). All study procedures were approved by the University of Rochester’s Institutional Review Board (RSRB00066408).

Study participation consisted of a baseline assessment, a 28-day real-time monitoring period including EMA and wrist actigraphy, and a final phone assessment at the end of the real-time monitoring period. The baseline assessment included interviews and questionnaires to assess background sociodemographic and diagnostic information, in addition to prevalence of STBs over the adolescents’ lifetime, past year, and past month. The baseline session concluded with an orientation to the EMA application, a prevention-oriented suicide risk assessment, and a review of the adolescent’s safety plan. Following the baseline assessment, adolescents completed 28 consecutive days of EMA and wrist actigraphy. Several types of EMA surveys from the larger project are relevant for the current study.

ICAM: Interval-contingent surveys were completed at a fixed time each morning (ICAM) within 2 h of waking up, which included a daily sleep diary (see Measures section). Participants completed an average of 16.04 sleep diaries each (*SD* = 8.06, Range = 3–28). The median ICAM completion time was 1 min 36 s (*SD* = 4 min 2 s).

SC: Signal-contingent (SC) or random surveys were completed 3–6 times each day. After receiving the survey prompt, adolescents were given 30 min to complete each survey. The window to complete surveys was based on each adolescent’s availability, which provided more time on some days than others (leading to a range of 3–6 available surveys). Adolescents were not prompted to complete surveys during weekday school hours. Although several SC prompts were offered each day, adolescents were only required to complete three SC surveys daily. During the EMA period, participants completed an average of 62.36 SC surveys (*SD* = 31.03, Range = 6-116). The median completion time for these surveys was 3 min 25 s. In these surveys, adolescents responded to prompts about anhedonia and suicidal thoughts (see Measures section).

Regarding compensation, adolescents and parents received $25/hour for the baseline assessment (up to a maximum of $75). For the 28-day monitoring period, adolescents were compensated with a $25 Amazon gift card for each week they completed at least 75% of the EMA surveys.

### Risk and Safety Monitoring During EMA Period

Responses to all suicide-related EMA questions were reviewed by the study team and steps were taken to ensure participant safety. A brief overview of the risk monitoring and management plan is provided below.

Suicide-related EMA responses were reviewed twice daily—once in the morning and once in the late afternoon/early evening—throughout the 28-day monitoring period. Risk thresholds for EMA items were established to create a standardized process for monitoring and assessing risk during the study. Since no predefined cutoffs exist for this kind of research, these thresholds were developed based on previous studies (e.g., Kleiman et al., 2017) and in consultation with the research and clinical teams. Suicide-related responses flagged for risk on the SC surveys included the following: suicide desire rating of ≥ 4 (Very intense), AND either suicide intent rating of ≥ 4 (Very strong), OR an inability to keep oneself safe rating of ≥ 3 (I’m not sure I can keep myself safe). In addition, any report of suicidal behavior—such as a suicide plan, aborted attempt, interrupted attempt, or suicide attempt—was flagged as high-risk.

If an adolescent was flagged for risk, the PI completed a risk assessment and followed up with the parent/caregiver as needed. To further safeguard adolescents and support families during the study, the research team collaborated with the medical center’s outpatient clinical team, including each adolescent’s outpatient clinician. This partnership ensured clear communication between the study staff, outpatient clinicians, and parents. Additionally, this collaboration provided parents with guidance on managing expectations and addressing risks related to suicide and clinical severity.

### Baseline Measures

#### Suicidal Thoughts and Behaviors (STBs)

The Columbia-Suicide Severity Rating Scale (Posner et al., 2011), which has been validated in adolescents (Gipson et al., 2015), was used to assess lifetime and recent suicide ideation, plans, and attempts. The lifetime, past year, and past month prevalence of STBs in the current study sample is presented in Table 1.

**Table 1:** Prevalence of suicidal thoughts and behaviors

Suicidal thoughts and behaviors:	% (*n*/*N*)
*Lifetime*	
Suicide ideation	100% (48/48)
Suicide attempt	85.4% (41/48)
Multiple attempts	61.0% (25/41)
*Past year*	
Suicide ideation	100% (48/48)
Suicide attempt	78.7% (37/47)
Multiple attempts	51.4% (19/37)
*Past month*	
Suicide ideation	91.7% (44/48)
Suicide attempt	31.3% (15/48)

#### Major Psychiatric Disorders

Major psychiatric disorders were assessed separately from both adolescents and parents using the Mini International Neuropsychiatric Interview for Children and Adolescents (MINI-Kid; Duncan et al., 2018). Current diagnoses were established by integrating reports from both adolescents and parents to provide a comprehensive characterization of the sample and are presented in Table 2.

**Table 2:** Prevalence of major psychiatric disorders

Major psychiatric disorders	% (*n*/*N*)
Anxiety disorder	93.5% (43/46)
Attention-deficit hyperactivity disorder	27.9% (12/43)
Bipolar disorder	6.5% (3/46)
Disruptive behavior disorder	25.0% (11/44)
Eating disorder	20.9% (9/43)
Major depressive disorder	82.6% (38/46)
Obsessive compulsive disorder	9.3% (4/43)
Posttraumatic stress disorder	20.0% (9/45)
Psychotic symptoms	7.0% (3/43)
Substance use disorder	8.7% (4/46)

*Current diagnoses were determined by integration of the adolescent and parent reports (obtained separately). Anxiety disorder includes any of the following current disorders: panic disorder, agoraphobia, social anxiety disorder, specific phobia, or generalized anxiety disorder; Attention-deficit hyperactivity disorder includes any of the following current subtypes: inattentive only, hyperactive/impulsive only, or combined; Bipolar disorder includes current bipolar I or II disorder; Disruptive behavior disorder includes current conduct disorder or oppositional defiant disorder; Eating disorder includes current anorexia nervosa or bulimia nervosa; Substance use disorder includes current alcohol use disorder or substance (drug) use disorder. Given time constraints, not all disorder modules were administered to all participants resulting in missing data

#### Medication History

At the baseline assessment, 45 adolescents (93.8% of the sample) reported taking at least one psychiatric medication. Of these, 37 (77.1%) were using some form of medication for sleep^2^. Specifically, 34 participants were taking prescription medications for sleep, with the most common being the off-label use of the antihistamine Hydroxyzine (66.7% of the total sample and 86.5% of those using any medication that could affect sleep). Additionally, six adolescents reported using over-the-counter sleep aids, including five who used melatonin and one who used Benadryl.

### EMA Measures

#### Total Sleep Time (Predictor)

A daily sleep diary, completed as part of the ICAM surveys each morning, was used to assess total sleep time (TST; i.e., total time spent asleep in minutes) consistent with the Consensus Sleep Diary (Carney et al., 2012). Shorter TSTs may indicate insufficient sleep duration, a commonly endorsed sleep problem (Short et al., 2013). This parameter was selected based upon results from Glenn et al. (2021) and Kearns et al. (2023) (see Glenn et al., 2021 for specifics regarding the calculation of this sleep parameter). In Glenn et al. (2021) and Kearns et al. (2023), shorter TST as assessed by sleep diaries, but not wrist actigraphy, predicted next-day suicidal thinking in some but not all models, and sleep diaries and wrist actigraphy assessing TST showed very strong measurement agreement (*r* = .85). Altogether, TST only via sleep diary predicted suicidal thinking in our prior study, however, there is strong measurement agreement between commonly used methods to assess TST.

#### Sleep Onset Latency (Predictor)

A daily sleep diary, completed as part of the ICAM surveys each morning, was also used to assess sleep onset latency (SOL), or time it takes to fall asleep in minutes, starting from when one intends to fall asleep, consistent with the Consensus Sleep Diary (Carney et al., 2012). Longer SOL may indicate greater difficulties falling asleep, another commonly endorsed sleep problem (Short et al., 2013). This parameter was selected based upon results from Glenn et al. (2021) and Kearns et al. (2023). In Glenn et al. (2021) and Kearns et al. (2023), greater SOL as assessed by sleep diaries, but not wrist actigraphy, predicted next-day suicidal thinking, and sleep diaries and wrist actigraphy assessing SOL showed moderate measurement agreement (*r* = .33). In sum, SOL only via sleep diary predicted suicidal thinking in our prior study, although sleep diary and wrist actigraphy demonstrated moderate convergence.

#### Consummatory and Anticipatory Anhedonia (Mediators)

Adolescents completed multiple SC surveys daily and reported on their momentary consummatory and anticipatory anhedonia. These items were developed based on prior self-report measure anhedonia items (in collaboration with an anhedonia researcher, Foti et al., 2018) and adapted for repeated, momentary assessments consistent with previous studies (Bakker et al., 2017; Wu et al., 2017). Consummatory anhedonia was assessed with a single item, “Think back to the most positive event that happened in the last couple of hours. How much did your mood change following this event?” Adolescents rated their current consummatory anhedonia on a scale of -2 (*mood worsened a great deal*) to 0 (*no mood change*) to + 2 (*mood improved a great deal*). Anticipatory anhedonia was assessed with a single item, “Think ahead to the most positive event that will happen over the next couple of hours. How much are you looking forward to this event?” Adolescents rated their current anticipatory anhedonia on a scale of 0 (*not at all*) to 2 (*somewhat*) to 4 (*extremely*). The structure of both items (couple of hours) was kept consistent to emphasize the key difference of time across the two items. For consummatory anhedonia, items were first recoded to a 0–4 scale, consistent with anticipatory anhedonia scale measurement. Next, for both consummatory and anticipatory anhedonia, items were reverse coded to aid with interpretation of effects, such that higher scores reflect greater anhedonia.

#### Suicidal Thoughts (Outcome)

Suicidal thoughts were assessed at multiple time points during the day via the SC surveys. Participants responded to three items adapted from prior EMA studies (Kleiman et al., 2017; Nock et al., 2009), that assessed current (at that moment) desire for life (a measure of passive ideation) on a scale from 1 (*very strong*) to 5 (*very weak*), suicide desire (a measure of active ideation) on a scale from 0 (*absent/no desire*) to 5 (*extremely intense*), and suicide intent on a scale from 0 (*absent/no desire*) to 5 (*extremely intense*). Across all items, higher scores indicated greater suicidal thoughts. We created a latent variable for suicidal thoughts consisting of these three items that were previously identified as items with high latent construct overlap, consistent with procedures used in prior studies (Glenn et al., 2022a), which will be referred to as suicidal thought intensity for the remainder of the manuscript.^3^  

### Data Preparation

For EMA, data were missing at the survey level (i.e., a survey was not completed) rather than at the item level (i.e., all items were completed in a single survey). If an ICAM survey (i.e., sleep diary) was missing, that day’s data were not included in the model (because the predictor was missing for that day). If a SC survey was missing (i.e., mediators: consummatory and anticipatory anhedonia, outcome: suicidal thoughts), other SC surveys from that day were included in the model. For lagged effect models (described further below), if all SCs were missing for that day (following an ICAM), or all but one (since we used two consecutive SCs for full temporal mediation), that day’s data was not included in the model. The configuration of our missing data involved a completely missing survey rather than a missing item from an otherwise complete survey. This type of missingness is expected in multi-level modeling (effectively only leading to unevenly spaced data) and cannot be imputed.

### Data Structure

To maximize available data and comprehensively characterize the time-varying mechanistic associations between sleep problems (TST, SOL), facets of anhedonia, and suicidal thoughts, we examined both contemporaneous (within the same EMA survey) and lagged (one EMA survey predicting the next EMA survey, within-day) effects in our models. TST and SOL were included as predictors at the day-level since these are assessed once daily in the morning and refer to sleep from the previous night. Facets of anhedonia and suicidal thoughts were measured at the momentary level multiple times per day. In both contemporaneous and lagged effect models, sleep problems temporally preceded anhedonia and suicidal thoughts. For contemporaneous models, facets of anhedonia and suicidal thoughts were measured at the same time-point using the same EMA survey. For lagged effect models, facets of anhedonia were measured at the time point prior to suicidal thoughts, within the same day, allowing us to examine full temporal mediation.

### Analytic Strategy

A series of multi-level structural equation models (MSEM) were conducted using the *lavaan* package (Rosseel, 2012) in *R* to test consummatory and anticipatory anhedonia as parallel mediators in the relationship between sleep problems (TST, SOL) and suicidal thoughts.

Our models examined both contemporaneous and lagged effects between mediators (consummatory and anticipatory anhedonia) and the outcome variable (suicidal thoughts). As such, a total of four parallel mediation models were analyzed. Two contemporaneous models included either TST or SOL as the predictor (day 1), anhedonia variables (day 2, time T) as parallel mediators, and suicidal thoughts (day 2, time T) as the outcome. Two lagged effect models included either TST or SOL as the predictor (day 1), anhedonia variables (day 2, time T) as parallel mediators, and suicidal thoughts (day 2, time T + 1, within-day) as the outcome.

MSEM is the preferred analytic approach since traditional multi-level mediation models may conflate the within and between effects in models (Preacher et al., 2010, 2011). MSEM can parse within and between effects of all variables, allowing for separate examination of effects which accounts for conflation concerns. Furthermore, MSEM can estimate direct and indirect effects in the same model. We estimated two structural equation models, a within-person model (our primary interest) and a between-person model. This allowed us to adequately partition the variance of within-person versus between-person effects. Since we are primarily interested in within-person, time-varying processes, we focused on the within-person component of the model.

In line with existing mediation approaches, we did not examine direct effects prior to testing the main mediation model. If there is theoretical rationale for examining mediation, testing direct effects first is not a requirement (Shrout & Bolger, 2002), as effect sizes may be small, or suppression may be possible.

## Results

Descriptive statistics for variables included in models are presented in Table 3. For each model below, both between-person and within-person effects were calculated and are reported separately in each figure. In text, we report on structural model fit and direct and indirect effects at the within-person level only, given that within-person effects are our primary focus.

**Table 3:** Descriptives for main study variables

Variable	M	SD	ICC range
Total sleep time (in minutes)	467.46	133.92	0.321-0.347
Sleep onset latency (in minutes)	41.40	53.02	0.435-0.451
Consummatory anhedonia	1.60	1.13	0.319-0.332
Anticipatory anhedonia	2.47	1.37	0.422-0.450
Suicidal thoughts– desire to live	2.70	1.21	0.718-0.720
Suicidal thoughts– suicide desire	0.65	1.01	0.452-0.464
Suicidal thoughts– suicide intent	0.29	0.63	0.431-0.464

ICC = intraclass correlation, *M* = mean, *SD* = standard deviation

### Measurement Model for Suicidal Thoughts Latent Variable

**Fig. 1:**
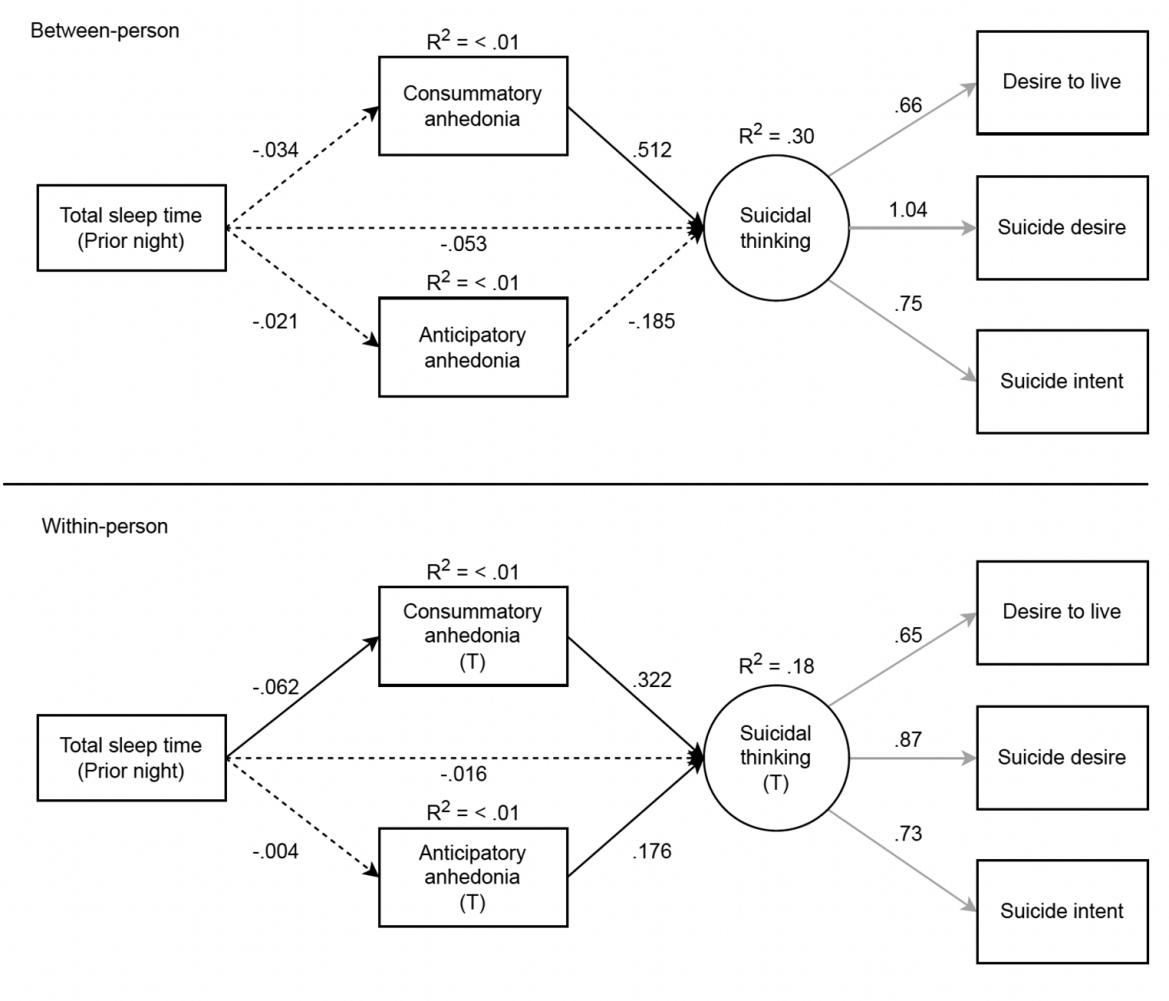
Results of the multi-level SEM testing consummatory and anticipatory anhedonia as mediators of the association between prior night total sleep time and suicidal thinking, which included 2,148 observations. Mediators and suicidal thinking (outcome) were measured at the same time point. T = momentary time-point over the next-day. Standardized estimates are shown. Black lines = structural model, grey lines = measurement model. Solid lines = significant at *p* <.05, dashed lines = not significant. The covariance between consummatory and anticipatory anhedonia variables at the within-person level are not shown for clarity but are reported here: B = .374, *p* <.001

**Fig. 2:**
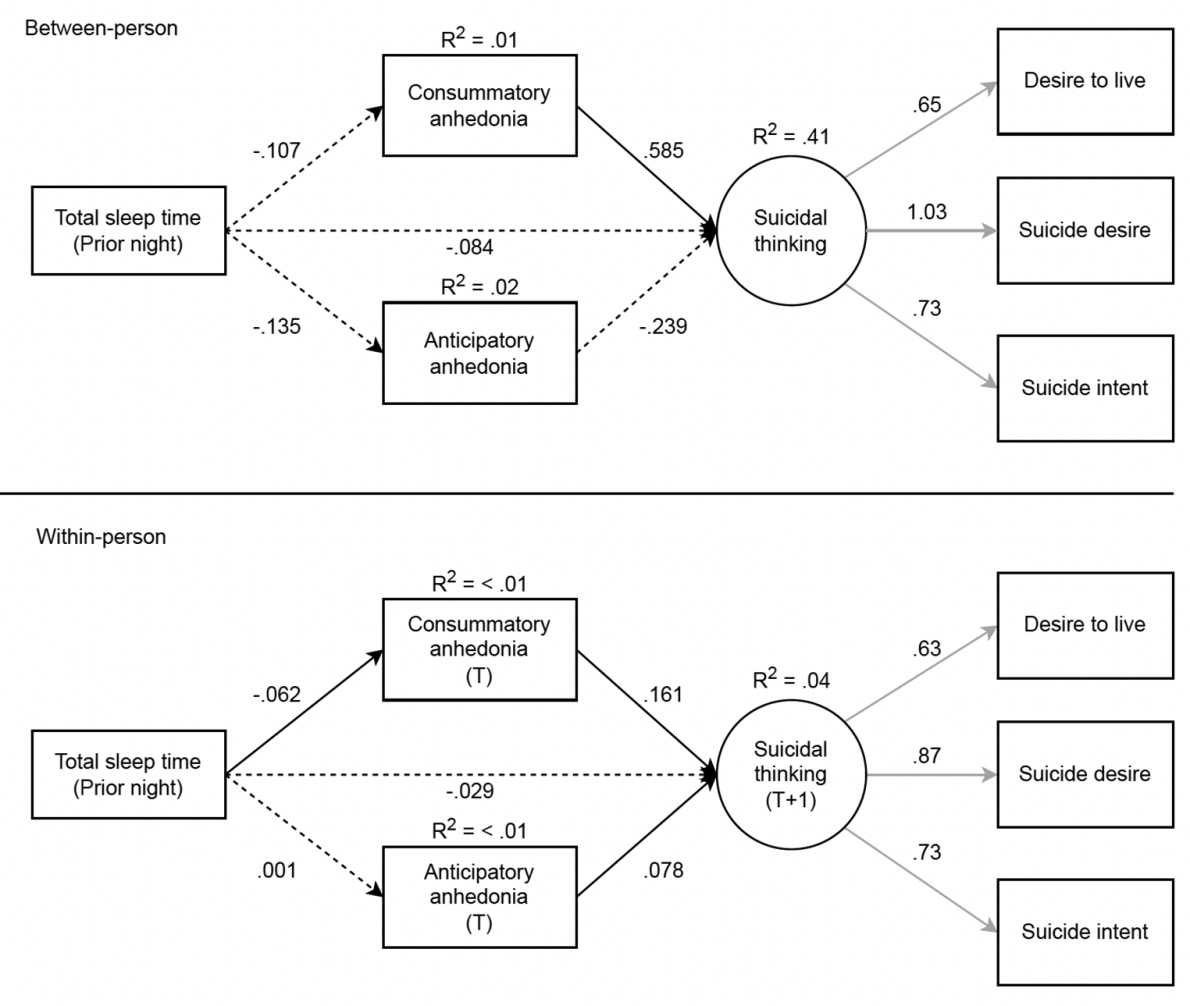
Results of the multi-level SEM testing consummatory and anticipatory anhedonia as mediators of the association between prior night total sleep time and suicidal thinking, which included 1,424 observations. Full temporal mediation examined with prior night total sleep time, anhedonia (next-day) and time T, and suicidal thinking (next-day), at time T + 1. T = momentary time-point 1 over the next-day, T + 1 = momentary time-point 2 over the next-day (subsequent EMA survey). Standardized estimates are shown. Black lines = structural model, grey lines = measurement model. Solid lines = significant at *p* <.05, dashed lines = not significant. The covariance between consummatory and anticipatory anhedonia variables at the within-person level are not shown for clarity but are reported here: B = .352, *p* <.001

**Fig. 3:**
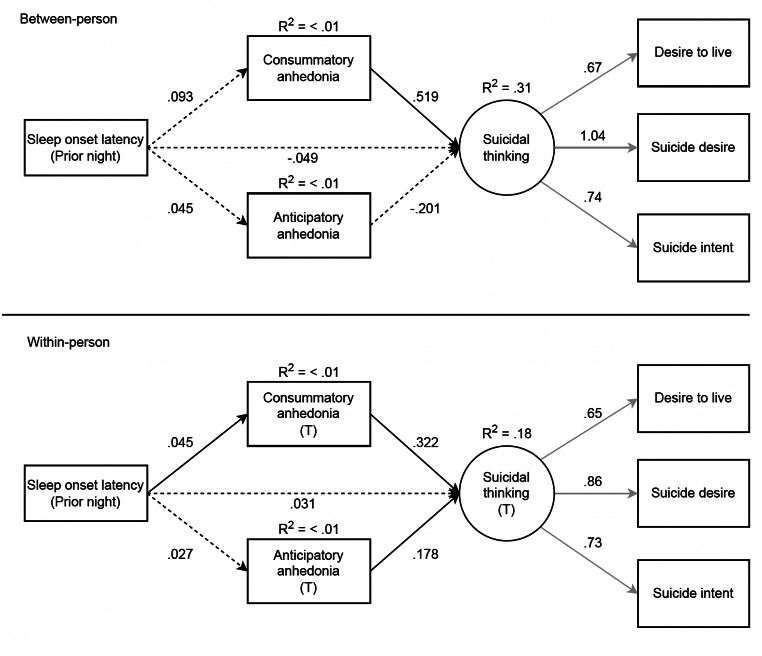
Results of the multi-level SEM testing consummatory and anticipatory anhedonia as mediators of the association between prior night sleep onset latency and suicidal thinking, which included 2,162 observations. Mediators and suicidal thinking (outcome) were measured at the same time point. T = momentary time-point over the next-day. Standardized estimates are shown. Black lines = structural model, grey lines = measurement model. Solid lines = significant at *p* <.05, dashed lines = not significant. The covariance between consummatory and anticipatory anhedonia variables at the within-person level are not shown for clarity but are reported here: B = .373, *p* <.001

**Fig. 4:**
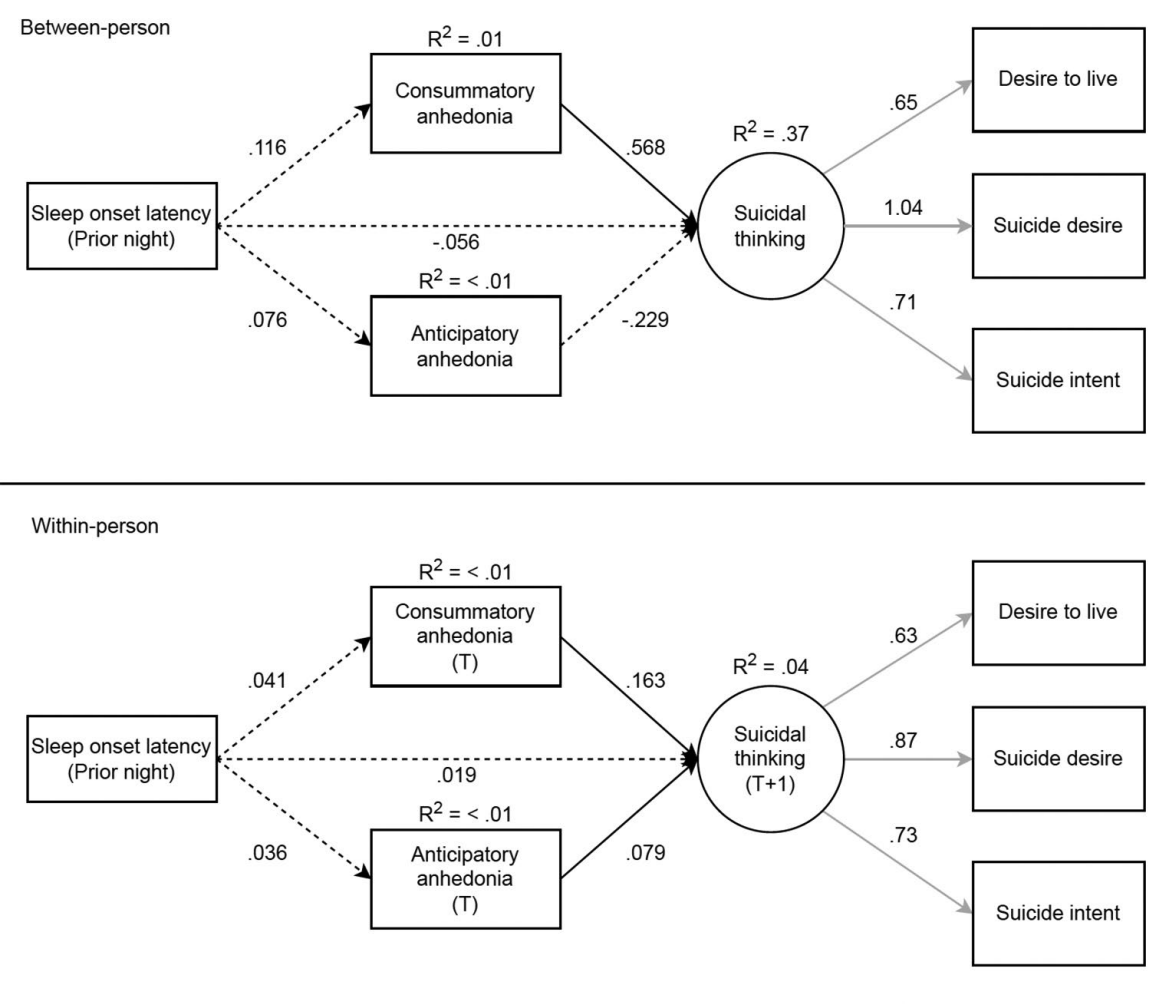
Results of the multi-level SEM testing consummatory and anticipatory anhedonia as mediators of the association between prior night sleep onset latency and suicidal thinking, which included 1,432 observations. Full temporal mediation examined with prior sleep onset latency, anhedonia (next-day) and time T, and suicidal thinking (next-day), at time T + 1. T = momentary time-point 1 over the next-day, T + 1 = momentary time-point 2 over the next-day (subsequent EMA survey). Standardized estimates are shown. Black lines = structural model, grey lines = measurement model. Solid lines = significant at *p* <.05, dashed lines = not significant. The covariance between consummatory and anticipatory anhedonia variables at the within-person level are not shown for clarity but are reported here: B = .353, *p* <.001

The latent suicidal thoughts variable was constructed from three observed variables assessing current intensity of suicidal thoughts, reflecting a saturated model. Thus, we are unable to assess model fit of the measurement model. Instead, we depict factor loadings for this latent variable across all structural models (see Figs. 1, 2, 3 and 4). Notably, the loadings in the measurement model were nearly identical to those in the structural models such that the observed suicidal thoughts variables significantly loaded onto the suicidal thoughts latent construct.

### TST Contemporaneous Model

The structural model fit was deemed acceptable (*CFI* = .93, *TLI* = .84, *RMSEA* = .09, 90% CI [.08,.10]). As shown in Fig. 1, there were significant direct effects in the within-person model between shorter TST and greater consummatory anhedonia, and greater consummatory anhedonia and greater suicidal thoughts. Further, consummatory anhedonia significantly mediated the association between TST and suicidal thoughts, such that shorter TST led to greater consummatory anhedonia, which in turn led to an increase in suicidal thoughts (B = −.02, *p* = .005). There were significant direct effects in the within-person model between greater anticipatory anhedonia and greater suicidal thoughts, however anticipatory anhedonia was not found to significantly mediate the association between TST and suicidal thoughts.

### TST Lagged Effect Model

The structural model fit was deemed acceptable (*CFI* = .94, *TLI* = .86, *RMSEA* = .07, 90% CI [.06,.09]). As shown in Fig. 2, there were significant direct effects in the within-person model between shorter TST and greater consummatory anhedonia, and greater consummatory anhedonia and greater next time-point suicidal thoughts. In this lagged effect model, consummatory anhedonia significantly mediated the association between TST and suicidal thoughts, such that shorter TST led to greater consummatory anhedonia, thereby increasing suicidal thoughts at the next within-day time-point (B = −.01, *p* = .03). There were significant direct effects in the within-person model between greater anticipatory anhedonia and greater next time-point suicidal thoughts, but anticipatory anhedonia did not significantly mediate the association between TST and suicidal thoughts in this lagged effect model.

### SOL Contemporaneous Model

The structural model fit was deemed acceptable (*CFI* = .93, *TLI* = .83, *RMSEA* = .09, 90% CI [.08,.10]). As shown in Fig. 3, there were significant direct effects in the within-person model between greater SOL and greater consummatory anhedonia, and greater consummatory anhedonia and greater suicidal thoughts. Moreover, consummatory anhedonia significantly mediated the association between SOL and suicidal thoughts, such that greater SOL led to greater consummatory anhedonia, which in turn led to an increase in suicidal thoughts (B = .015, *p* = .04). There were significant direct effects in the within-person model between greater anticipatory anhedonia and greater suicidal thoughts, however anticipatory anhedonia was not found to significantly mediate the association between SOL and suicidal thoughts.

### SOL Lagged Effect Model

The structural model fit was deemed acceptable (*CFI* = .94, *TLI* = .85, *RMSEA* = .08, 90% CI [.06,.09]). As shown in Fig. 4, in the within-person model, there were significant direct effects between greater consummatory anhedonia and greater next time-point suicidal thoughts, and greater anticipatory anhedonia and greater next time-point suicidal thoughts. No significant indirect mediation effects were observed in this lagged effect model.

## Discussion

The current study examined anhedonia as a potential mechanism linking sleep problems and suicidal thoughts in high-risk adolescents using an intensive longitudinal study design. Overall, the results of this study provide novel, albeit preliminary, evidence for anhedonia as a mechanism in the sleep-STBs association. The main takeaways from our findings are described in turn.

This study significantly extends prior research on the sleep problems-STB association by investigating *how*,* or the processes by which*, sleep problems confer risk for STBs. Our findings revealed *consummatory* anhedonia significantly mediated the relation between sleep problems and suicidal thoughts. More specifically, shorter TST and greater SOL were significantly related to greater consummatory anhedonia, which was subsequently related to greater suicidal thought intensity. These results suggest that less sleep (i.e., shorter TST) or greater difficulties falling asleep (i.e., longer SOL) resulted in diminished capacity to experience in-the-moment pleasure the next day. This diminished or decreased capacity to experience pleasure was related to greater suicidal thought intensity. In all but one of our models (the SOL lagged effect model), consummatory anhedonia significantly mediated the association between sleep problems and suicidal thoughts. Taken together, our study suggests sleep problems were associated with suicidal thought intensity through consummatory anhedonia.

Interestingly, we did not find evidence for anticipatory anhedonia as a mediator between sleep problems and suicidal thoughts in our study. Our null findings may be attributable to a few factors. For one, it may be that sleep problems are more robustly associated with consummatory anhedonia (in-the-moment experience of pleasure in the presence of reward) rather than anticipatory anhedonia (prediction of pleasure from future reward). One prior study preliminarily substantiates this possibility, indicating that consummatory anhedonia was more globally associated with sleep problems both at baseline and over the course of a daily diary monitoring period (Wieman et al., 2022). It may be that consummatory anhedonia reflects an aspect of diminished reward responsiveness that is inherently more sensitive to the effects of sleep problems. Importantly, this hypothesis posits that alterations to the consummatory aspects of reward processing are not the direct result of, but rather influenced by, prior night’s sleep problems. Prospective longitudinal research would be needed to fully ascertain the cause of alterations to reward processing.

Our main findings are generally consistent with prior research evidencing a link between sleep problems and anhedonia in adolescents (Burani et al., 2021; Casement et al., 2016). As previously mentioned, our mediation results were specific to consummatory anhedonia, suggesting a unique relation between sleep problems and this facet of anhedonia. Similar to our findings, a recent daily diary study in adults found that shorter TST and longer SOL predicted decreased consummatory reward responsiveness (Wieman et al., 2022). In contrast to our null findings, Wieman et al., 2022 found that shorter total sleep time predicted blunted anticipatory reward responsiveness. This divergence with regards to anticipatory anhedonia may also reflect differences in measurement of this construct in our study as compared with prior studies. For instance, Wieman et al., 2022 asked participants to rate how “enjoyable, interested, and/or motivated” they expected to feel in response to a future experience, whereas our item assessed the extent to which participants were “looking forward” to a future event. To date, only a handful of studies have assessed anticipatory anhedonia using a real-time monitoring approach (Bakker et al., 2017; Wieman et al., 2022; Wu et al., 2017) rendering this an emerging research area. Furthermore, the majority of past research on anhedonia in clinical populations in the context of depression has focused on the consummatory aspect of anhedonia (Treadway & Zald, 2011), and depression has been more closely linked with deficits in consummatory anhedonia rather than anticipatory anhedonia (Foti et al., 2018). It may also be that our anticipatory anhedonia EMA item was imprecise in its assessment of this construct. Altogether, our findings suggest that future research should continue to delineate facets of anhedonia to comprehensively account for differential associations with STBs.

Although not a primary aim of the current study, anticipatory and consummatory anhedonia were significantly associated with suicidal thought intensity at the within-person level, and consummatory anhedonia was significantly related to suicidal thought intensity at the between-person level. Results from our within-person analyses demonstrate that an individual’s anticipatory and consummatory anhedonia change over time and that these processes are significantly associated with suicidal thought intensity. Our findings lend further support to the overall relationship between anhedonia and suicidal thoughts among youth (e.g., Auerbach et al., 2015; Yang et al., 2021, 2020b) and extends this to the within-person level.

### Implications of Findings from a Developmental Perspective

Findings from this study are particularly important given the limited research on the sleep-STB association in youth. Adolescence is a developmental period characterized by widespread biological and psychosocial changes resulting in significant changes in sleep patterns and circadian rhythm timing which may contribute to an increase in difficulties with sleep (Harvey et al., 2018). Our assessment of sleep problems in the current study centered on shorter TST and longer SOL, which are typically conceptualized as components of insomnia (Edinger et al., 2004). However, it may be that these sleep problems reflect alterations in sleep timing, which may be uniquely associated with suicidal thoughts during this developmental stage. For instance, if adolescents attempt to initiate sleep at a time that is misaligned with their circadian rhythm preference, they may experience difficulties falling asleep reflected by a longer SOL. These delays in sleep timing may also result in shorter TST (Gradisar et al., 2011; Williamson et al., 2019). This is an important distinction for future research to uncover, as this will bolster our understanding of sleep problems as a developmentally informed risk factor for suicide in adolescence and may have direct implications for clinical intervention.

### Clinical Implications

Preliminary results point to sleep problems and anhedonia as potential proximal, time-varying risk factors for STBs, and importantly, these risk factors may be amenable to intervention. For example, evidence-based sleep interventions such as cognitive behavioral therapy for insomnia (Blake et al., 2017; Clarke et al., 2015) or the transdiagnostic sleep and circadian intervention (TranS-C; Dong et al., 2020; Harvey et al., 2018) are therapeutic approaches that target a range of sleep and circadian difficulties in youth. Although these sleep interventions have broadly shown promise in reducing sleep problems in youth (Blake et al., 2017; Clarke et al., 2015; De Bruin et al., 2015; Dong et al., 2020), and in some cases depression and anxiety symptoms (Blake et al., 2017; Clarke et al., 2015), no published research to date has tested sleep interventions in youth with STB outcomes (Blake & Allen, 2020).

Moreover, interventions targeting anhedonia may be beneficial for adolescents who experience low levels of positive affect or enjoyment, and low levels of motivation to engage in pleasurable activities. Existing interventions targeting anhedonia stem from approaches to reduce depressive symptoms, given that anhedonia is a cardinal symptom of depression (American Psychiatric Association, 2013). One such intervention, behavioral activation, has accumulating evidence indicating its effectiveness in reducing depressive symptoms in adolescents (McCauley et al., 2016; Ritschel et al., 2016; Tindall et al., 2017). Importantly, behavioral activation centers on increasing exposure to and engagement with rewarding activities (Webb et al., 2023), highlighting its utility for adolescents with anhedonia.

### Limitations and Future Directions

The findings from the current study should be viewed in the context of several important limitations. First, while the study had sufficient power for within-person analyses and our multi-level SEM model included person-level means, we lacked the power to include between-person covariates and conduct between-person analyses to investigate specific individual differences in overall effects. As a result, we were unable to assess how demographic factors, such as gender identity and race/ethnicity, might moderate sleep-suicide associations. Rates of suicide risk among racial/ethnic (Goldstein et al., 2021; Xiao et al., 2021) and gender/sexual (Liu et al., 2020b; Pollitt & Mallory, 2021) minority youth are high and increasing. Moreover, emerging research indicates greater sleep problems among racial/ethnic (El-Sheikh et al., 2022; Yip et al., 2022) and gender/sexual minority youth (Levenson et al., 2020), reflecting an imperative need to address significant mental health disparities impacting diverse youth by conducting studies designed specifically to understand the sleep-suicide risk association in diverse populations (Goldstein et al., 2021). Further, the sample consisted of participants who were predominantly female and white, limiting the generalizability of our findings to more diverse samples. It should be noted, our findings are preliminary and require replication in larger, well-powered samples.

Second, this study is among the first to examine mechanisms linking sleep problems and STBs (Hamilton et al., 2023), however, anhedonia is just one potential mechanism that may be implicated in the sleep-suicide association. Other potential cognitive and affective mediators, such as emotion dysregulation (Andrews & Hanna, 2020; McCall & Black, 2013), are worthwhile avenues for future mechanistic investigations and may uniquely contribute to STB risk. Further, the primary objective in the current study was to extend our understanding of how TST and SOL *specifically* may be linked to suicidal thoughts over the short-term. Interestingly, there is emerging evidence that additional sleep problems such as nightmares (Glenn et al., 2021) and sleep quality (Hamilton et al., 2023) may be important to understanding STB risk. A limitation of the current study is the narrow scope of sleep parameters included, and future research would benefit from a more comprehensive assessment of sleep problems.

Third, the current study was limited in its examination of daily mood symptoms (i.e., depression symptoms), that may impact the sleep-anhedonia-STB association. Future research should be undertaken to explore the extent of the influence of mood symptoms, and other potentially relevant covariates, on the sleep-anhedonia-STB association.

Fourth, growing research suggests that examining the nonlinear relation between total sleep time and mental health outcomes, including STBs, may be relevant. A recent systematic review uncovered robust curvilinear associations between sleep duration and STB outcomes (Chiu et al., 2018). In future studies, specialized analytic approaches to further understand these nonlinear associations using temporally sensitive assessments in high-risk psychiatric adolescents is warranted to clarify the sleep-STB association.

Finally, emerging research indicates that studies should focus on how sleep over multiple, consecutive days may impact risk for STBs. Importantly in youth, cumulative sleep debt, or restricted sleep over multiple consecutive days with limited opportunity to catch up on sleep, may have a deleterious effect on next-day affect. Furthermore, variability in daily sleep patterns that may arise from delays in sleep timing due to circadian preference mismatches and restricted sleep periods, both of which are implicated in psychopathology broadly (Gregory & Sadeh, 2016), may be another promising sleep parameter to investigate. Indeed, a recent systematic review underscores the critical need for future research addressing sleep variability and timing from a developmentally informed perspective (Becker et al., 2017).

## Conclusion

In conclusion, this study’s findings provide an innovative examination of anhedonia as a mechanism linking sleep problems and STBs among high-risk adolescents. Specifically, this is the first study to examine sleep problems, anhedonia, and STBs using a temporally sensitive methodological approach in clinically high-risk youth. Most notably, this study found preliminary support for consummatory anhedonia as a mechanism linking sleep problems and STBs in high-risk youth. Ultimately, findings from this research using an intensive longitudinal design could help inform proximal, modifiable targets for intervention to reduce STB risk in youth.

## Data Availability

The data that support the findings of this study are available on request from the corresponding author, KKP. The data are not publicly available due to their containing information that could compromise the privacy of research participants.

## Footnotes

1 The goal of the prior study was to test direct relationships between a wide range of sleep problems and suicidal thoughts. Specifically, various sleep parameters were entered into a machine learning model to determine which parameters were most robustly associated with suicidal thoughts. There was no mechanistic investigation to clarify *how* sleep problems are associated with suicidal thoughts.

2 Among the 37 participants who reported at baseline using medications that could influence sleep, only 24 (65%) indicated using these medications during the EMA phase. This discrepancy raises concerns about the reliability of daily reporting on sleep medication use. Since we cannot confirm whether sleep medications were taken on days they were not reported, we excluded sleep medications from the study’s models.

3 Unlike our prior study, (Glenn et al., 2022a), the safety suicidal thoughts item was dropped from the latent variable due to poor loading onto the latent factor.
